# The Brief Case: Tastes of travel—intestinal importation

**DOI:** 10.1128/jcm.01655-25

**Published:** 2026-05-13

**Authors:** Lydia M. Mendoza, Blaine Mathison, Aliya Rehman, Paul M. Luethy

**Affiliations:** 1 Department of Pathology, University of Maryland School of Medicine 12264, Baltimore, Maryland, USA; 2 ARUP Laboratories 33294, Salt Lake City, Utah, USA; 3 Department of Pathology, University of Utah 161530, Salt Lake City, Utah, USA; 4 Department of Medicine, Division of Infectious Diseases, R. Adams Cowley Shock Trauma Center, University of Maryland School of Medicine 12264, Baltimore, Maryland, USA; Endeavor Health, Evanston, Illinois, USA

**Keywords:** ova and parasite examination, tapeworm, foodborne parasite, cestode, taeniasis, *Taenia saginata*

## CASE

A 32-year-old, otherwise healthy male living in Maryland was transferred to the Shock Trauma Center at the University of Maryland Medical Center after being struck by a motor vehicle. The patient presented with polytrauma with extensive fractures. The patient experienced hypoxia and a persistent air leak consistent with puncture of the lung parenchyma. Mechanical ventilation was instituted for respiratory support. Six days later, the patient underwent surgery to correct fractures to the ribs and remove damaged portions of the lung.

Since the first day of admission, the patient exhibited low-grade fever and leukocytosis. These clinical characteristics suggested the possibility of a respiratory infection, though the overall clinical picture was more consistent with atelectasis. A sputum specimen was sent for culture 3 days after surgery. The culture was positive for *Streptococcus pneumoniae*, and a short course of ampicillin-sulbactam was prescribed. The patient improved quickly and was extubated.

While admitted to shock trauma, the patient expelled a long and segmented body resembling a tapeworm in stool. The potential worm segment was sent to Associated Regional and University Pathologists Laboratories in ethanol for macroscopic examination and staining, and a stool specimen was sent for an ova and parasite (O&P) examination. A thorough history was obtained by the infectious disease service, and it was found that the patient was born in the United States, enjoys hiking, and recently drank water from the Shenandoah River. The patient reported that he does not eat pork, prefers meat to be cooked well done, and had last eaten sushi about 6 months prior. Travel history included trips to Colombia and Ethiopia within the past 3 years. A head computed tomography (CT) scan was relatively unremarkable, and no clinical signs of neurocysticercosis were observed. The patient had normal B12 levels without evidence of anemia or eosinophilia.

**Fig 1:**
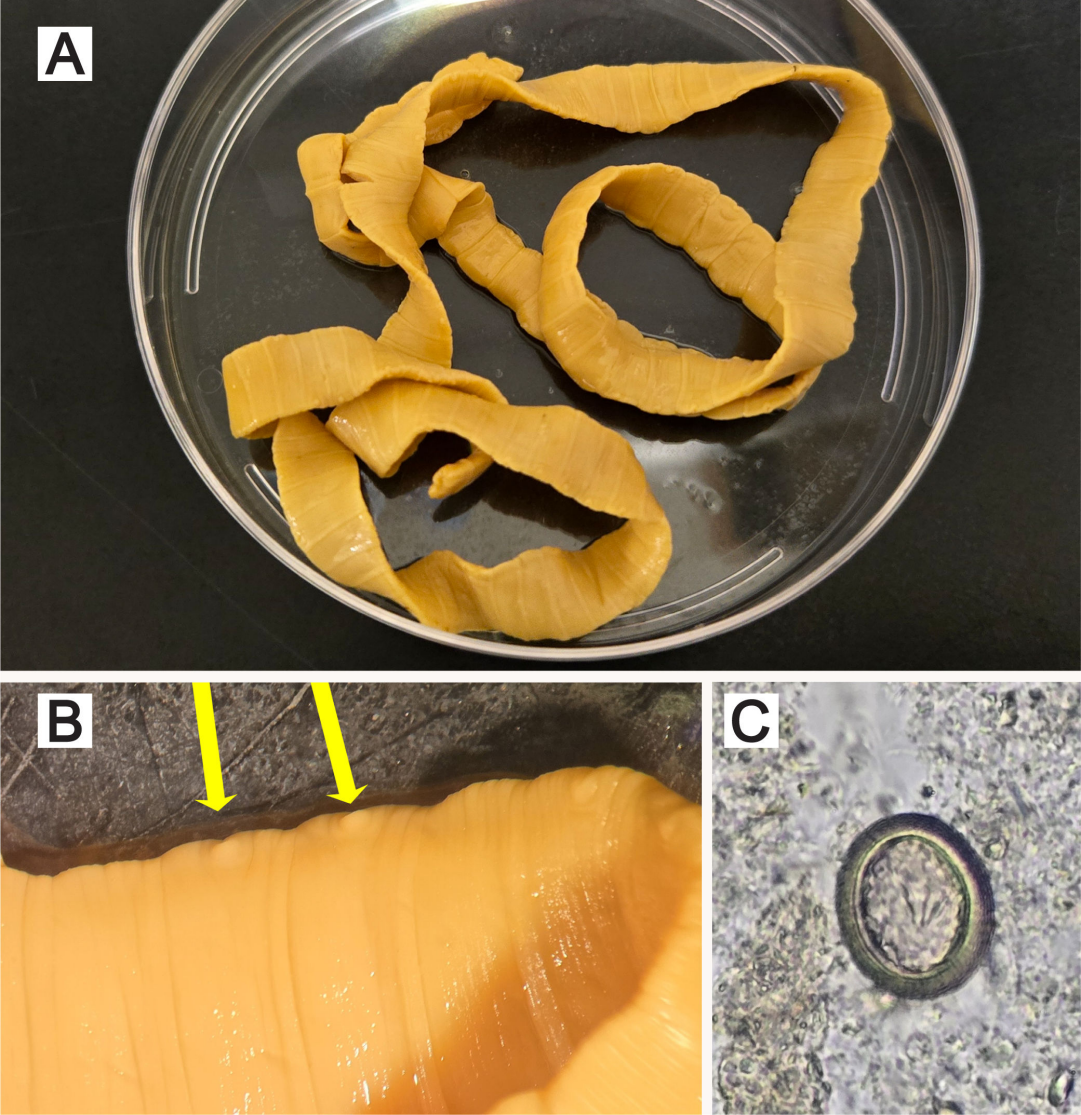
(**A**) Tapeworm strobila submitted for examination. (**B**) Close-up view of the proglottids, which are wider than they are long and feature lateral genital pores highlighted by the yellow arrows. (**C**) Egg recovered from the stool specimen. The egg is slightly oblong and has a thick radially striated shell. Three of six internal hooklets are visible in this plane.

Following examination, the specimen was determined to be a long section of tapeworm totaling 66.5 cm in length. Some regions of the tapeworm proglottids were wider than long, while some regions had proglottids slightly longer than wide (Fig. 1A). The expelled tapeworm did not have a scolex, indicating that it likely remained attached to the patient’s intestinal wall. Upon further observation, a single genital pore was noted on one side of each proglottid (Fig. 1B). The proglottids did not have a fully developed reproductive system, making them immature. As such, clearing and India ink injection techniques were not performed. Eggs were observed in a concentrated wet mount of stool during the O&P examination. Eggs were 35–40 μm in diameter, circular, and had a thick striated exterior shell (Fig. 1C).

The patient eventually revealed that he had eaten a raw beef delicacy while in Ethiopia. Therefore, based on patient history, the specimen was identified as *Taenia* species, likely *Taenia saginata*, known as the beef tapeworm. The patient was treated with a single 600 mg dose of praziquantel. Stool re-examination is recommended 3 months post-treatment to document infection clearance; however, this patient was lost to follow-up.

## DISCUSSION

Taeniasis is an intestinal infection caused by tapeworms of the *Taenia* genus. *Taenia* belongs to the class Cestoda, made up of parasitic intestinal tapeworms, including other familiar genera: *Dibothriocephalus* (and related, formerly included in *Diphyllobothrium*), *Rodentolepis* (formerly in *Hymenolepis*), and *Echinococcus*. These parasites require both intermediate and definitive hosts, with humans often serving as the definitive host (1). All tapeworms use an organ called the scolex to attach to a host’s intestinal wall. The tapeworm body, known as the strobila, is made up of a chain of segments that protrudes from the scolex and grows longer as the worm matures. Each segment, known as a proglottid, contains male and female reproductive organs (2). Tapeworm infections can be asymptomatic and are often discovered when proglottids are passed in stool.

Infection with a tapeworm of the genus *Dibothriocephalus*, known as diphyllobothriasis, and taeniasis are contracted orally with consumption of raw or undercooked meat, and these infections can last for several years. Untreated *Taenia solium* infections can persist for 2–3 years, and *T. saginata* can persist for over 20 years (3, 4). *Dibothriocephalus* spp. infection can persist for up to 25 years (5). Given the patient travel history and dietary practices, it was most likely that he acquired a tapeworm of the *Dibothriocephalus* or *Taenia* genera. Diphyllobothriasis cases have been reported worldwide; in North America, *Dibothriocephalus nihonkaiense* infections occur in the Pacific Northwest, and *Dibothriocephalus latus* infections occur in the Upper Midwest and Great Lakes regions (6–8). With *Dibothriocephalus*, freshwater fish are the intermediate and paratenic hosts, and humans who consume raw or poorly cooked fish serve as a definitive host (1). Some patients infected with *Dibothriocephalus* develop vitamin B12 deficiency, resembling pernicious anemia (9). Sushi consumption raised initial suspicion for a *Dibothriocephalus* infection despite normal bloodwork. Notably, this patient did not have eosinophilia, which is commonly associated with certain helminth infections (10). Among travelers returning from sub-Saharan Africa, helminths frequently linked to eosinophilia include *Schistosoma mansoni*, *Schistosoma haematobium*, *Trichinella* spp., and *Strongyloides stercoralis* (10).

There are two *Taenia* species that could have infected the patient, given his travel history: *T. saginata*, known as the beef tapeworm, and *T. solium*, known as the pork tapeworm. As suggested by their names, *T. saginata* exclusively infects cows, and *T. solium* exclusively infects pigs. A third species, *Taenia suihominis*, is endemic to East Asia and therefore unlikely to be the etiologic agent (11, 12). The life cycle of *Taenia* begins when humans ingest raw or undercooked beef or pork containing encysted larvae known as cysticerci. Cows or pigs acquire cysticerci through ingestion of *Taenia* eggs in areas contaminated with human stool. Upon ingestion, the eggs hatch and penetrate the intestinal wall and enter the bloodstream or lymphatic system, where they are filtered out into striated muscle and remain in an encysted state (1). For *T. solium*, there is a risk of self-reinfection upon ingestion of tapeworm eggs. When *T. solium* eggs from human stool are ingested by a human or pig, they hatch in the duodenum or jejunum, and the released oncospheres penetrate the intestinal wall and are carried throughout the body (1). The oncospheres are filtered out in subcutaneous tissues, intramuscular tissues, the eyes, and the brain (1). Neurocysticercosis is a condition when the *T. solium* cysticerci are found within the central nervous system, and symptoms include abnormal behavior, meningoencephalitis, transient paresis, seizures, and visual issues (1). The prognosis of neurocysticercosis is poor when the cysticerci proliferate and cannot be removed surgically or medically treated (1). Differentiating between a case of *T. solium* and *T. saginata* is important because of the complications associated with *T. solium* infection. If there is concern for *T. solium* infection, an assessment for neurocysticercosis should be performed by CT or magnetic resonance imaging scan. *T. solium* IgG enzyme-linked immunotransfer blot (EITB) is the recommended serologic assay for confirmatory testing (13). The enzyme-linked immunosorbent assay (ELISA) is also used for detection of *T. solium* neurocysticercosis, though it is less sensitive than EITB. Additionally, *T. solium* IgG measured by ELISA has previously led to false positives in individuals with other helminth infections through detection of cross-reactive antibodies (14).

*T. saginata* is more common than *T. solium* within the United States (2). Autochthonous *T. saginata* cases are rare; however*,* there have been limited outbreaks within American cattle farms (15). *T. solium* is most common in Central and South America and much of Asia, including South, East, and Southeast Asia (16). *T. saginata* transmission occurs in Asia and Latin America and is highly prevalent in the Middle East and North Africa (17). Several regional delicacies that incorporate raw or undercooked beef can serve as sources of *T. saginata* infection. In this case, the patient consumed *kitfo*, an Ethiopian dish of minced raw beef with seasoned spices and clarified butter. Another commonly consumed Ethiopian dish, *kurt*, is prepared from strips of raw beef. Similar raw beef preparations are popular in other regions, including steak tartare (Europe), *kibbeh nayyeh* (Levant), *crudo alemán* and *bistec alemán* (Chile), and *carpaccio* (Italy).

Although geography and reported dietary practices can guide diagnosis, definitive identification often relies on the O&P examination. An individual infected by a tapeworm will not always expel eggs or proglottids in a single stool specimen, potentially necessitating repeat examinations. Stool specimens should be placed in a fixative such as 10% formalin or alcohol-based fixatives used in single-vial collection systems prior to examination (18). Here, we analyzed a long section of strobila submitted in ethanol for macroscopic examination. The parasite examination typically involves clearing the mature proglottids with lactophenol and staining with India ink to visualize uterine structures, which are species specific (18). *Dibothriocephalus* tapeworms have a uterus located in the center of each gravid proglottid, with a characteristic rosette-shaped structure (2). In contrast, *T. saginata* and *T. solium* exhibit branched uterine structures that can be differentiated by the number of branches coming off of one side of the central uterine stalk. *T. saginata* has 12–30 uterine branches per side, while *T. solium* has 7–13. In this case, the expelled proglottids lacked fully developed uterine structures, thus complicating identification. The expelled proglottids were wider than they were long, which is characteristic of *Dibothriocephalus* (and related, Fig. 1A) (2). However, immature *Taenia* proglottids can appear shortened. An experienced parasitologist would recognize the lateral genital pores present on the proglottids, which are characteristic of *Taenia* species (Fig. 1B) (19). *Dibothriocephalus* has instead a central genital pore (5). O&P examination revealed *Taenia* eggs, which lack the operculum present in *Dibothriocephalus* (2). The eggs of *T. saginata* and *T. solium* are indistinguishable, measuring between 31 and 43 μm (2), with a thick, striated shell, enclosing a mature oncosphere with six refractile hooklets. The scolex of *T. saginata* is unarmed and has no hooks, while *T. solium* has many small hooks on its scolex used for attachment (2). Given the patient travel history and known consumption of raw beef, we identified the tapeworm as most likely *Taenia saginata*.

Here, we present an unexpected case of taeniasis in a Maryland trauma center that was likely acquired abroad. Taeniasis is uncommon in the United States, and tapeworm species identification may be required for adequate care, such as ascertaining the risk of cysticercosis. Species identification often requires collaboration with reference laboratories as the expertise and equipment necessary may not be present in most hospitals. In this case, we examined tapeworm eggs and a tapeworm section without fully developed uterine structures, which led us to rely heavily on patient history for diagnosis. It should be noted, however, that patient history, such as dietary habits or travel history, may be inaccurate due to recall bias or fear of judgment.

## SELF-ASSESSMENT QUESTIONS

1. Which method is best for differentiating between *T. solium* and *T. saginata* infections?
  - *T. solium* serum IgG ELISA
  - Egg morphology
  - Structure of lateral uterine branches (visualized after clearing or with India ink)
  - Length of tapeworm
2. Infection with which tapeworm species can result in cysticercosis?
  - *Taenia saginata*
  - *Taenia solium*
  - *Dibothriocephalus latus*
  - *Echinococcus* species
3. How can humans become infected with *T. saginata*?
  - Through autoinfection by ingestion of eggs produced by an intestinal tapeworm
  - Through consumption of raw or undercooked fish
  - Through consumption of raw or undercooked beef
  - Through consumption of raw or undercooked pork

## ANSWERS TO SELF-ASSESSMENT QUESTIONS

1. Which method is best for differentiating between *T. solium* and *T. saginata* infections?
  - *T. solium* serum IgG ELISA
  - Egg morphology
  - Structure of lateral uterine branches (visualized after clearing or with India ink)
  - Length of tapeworm
  Answer: c. There are more lateral uterine branches in the proglottids of *T. saginata* (12–30) than *T. solium* (7–13). The eggs of these species are identical, and tapeworm length cannot be relied upon for identification as the worm is usually passed in sections. Serum IgG ELISAs have been shown to detect cross-reactive antibodies from other helminth infections and thus are not the most reliable option for diagnosis of a *T. solium* infection.
2. Infection with which tapeworm species can result in cysticercosis?
  - *Taenia saginata*
  - *Taenia solium*
  - *Dibothriocephalus latus*
  - *Echinococcus* species
  Answer: b. *T. solium* is the only tapeworm species that can cause cysticercosis, a condition where eggs are ingested in food or fomites contaminated with human feces, and the larval form of the tapeworm enters the bloodstream and forms a cyst within human tissue.
3. How can humans become infected with *T. saginata*?
  - Through autoinfection by ingestion of eggs produced by an intestinal tapeworm
  - Through consumption of raw or undercooked fish
  - Through consumption of raw or undercooked beef
  - Through consumption of raw or undercooked pork
  Answer: c. *T. saginata* infects cattle as its intermediate host and is thus only found in beef, not fish or pork. It can only be acquired by consuming raw or undercooked beef.

**TAKE-HOME POINTS**

- *T. saginata* is acquired through consumption of raw or undercooked beef, while *T. solium* is acquired by consumption of raw or undercooked pork.
- *T. saginata* does not cause cysticercosis, which makes it important to differentiate between *T. saginata* and *T. solium* infections.
- Diagnosis of taeniasis or diphyllobothriasis often relies upon ova and parasite exam of stool or gross microscopy of an adult worm by an experienced parasitologist. One will not always have a complete or mature tapeworm specimen with a scolex and mature proglottids for definitive diagnosis.
- Praziquantel is the most effective treatment for *T. saginata* infection.
- Patients should be examined for the presence of eggs or proglottids in stool 3 months after initial treatment to check for clearance of infection.
